# The Diversity of Astrocyte Activation during Multiple Sclerosis: Potential Cellular Targets for Novel Disease Modifying Therapeutics

**DOI:** 10.3390/healthcare11111585

**Published:** 2023-05-29

**Authors:** Konstantinos Barmpagiannos, Paschalis Theotokis, Steven Petratos, Maurice Pagnin, Ofira Einstein, Evangelia Kesidou, Marina Boziki, Artemios Artemiadis, Christos Bakirtzis, Nikolaos Grigoriadis

**Affiliations:** 1 Laboratory of Experimental Neurology and Neuroimmunology, Second Department of Neurology, Aristotle University of Thessaloniki, 54621 Thessaloniki, Greece; mparmpak@auth.gr (K.B.); ptheotokis@auth.gr (P.T.); kesidoue@auth.gr (E.K.); bozikim@auth.gr (M.B.); 2 Department of Neuroscience, Central Clinical School, Monash University, Melbourne, VIC 3004, Australia; steven.petratos@monash.edu (S.P.); maurice.pagnin@monash.edu (M.P.); 3 Department of Physical Therapy, Faculty of Health Sciences, Ariel University, Ariel 40700, Israel; ofirae@ariel.ac.il; 4 Faculty of Medicine, University of Cyprus, Nicosia 2029, Cyprus; artemiadis.artemios@ucy.ac.cy

**Keywords:** astrocytes, reactivity, reactive astrocytes, transcriptional subsets, functional subsets, myelination, multiple sclerosis, multiple sclerosis therapies, disease-modulating therapies

## Abstract

Neuroglial cells, and especially astrocytes, constitute the most varied group of central nervous system (CNS) cells, displaying substantial diversity and plasticity during development and in disease states. The morphological changes exhibited by astrocytes during the acute and chronic stages following CNS injury can be characterized more precisely as a dynamic continuum of astrocytic reactivity. Different subpopulations of reactive astrocytes may be ascribed to stages of degenerative progression through their direct pathogenic influence upon neurons, neuroglia, the blood-brain barrier, and infiltrating immune cells. Multiple sclerosis (MS) constitutes an autoimmune demyelinating disease of the CNS. Despite the previously held notion that reactive astrocytes purely form the structured glial scar in MS plaques, their continued multifaceted participation in neuroinflammatory outcomes and oligodendrocyte and neuronal function during chronicity, suggest that they may be an integral cell type that can govern the pathophysiology of MS. From a therapeutic-oriented perspective, astrocytes could serve as key players to limit MS progression, once the integral astrocyte–MS relationship is accurately identified. This review aims toward delineating the current knowledge, which is mainly focused on immunomodulatory therapies of the relapsing–remitting form, while shedding light on uncharted approaches of astrocyte-specific therapies that could constitute novel, innovative applications once the role of specific subgroups in disease pathogenesis is clarified.

## 1. Introduction

Neuroglial cells constitute the most numerous and heterogeneous group of cells in gray and white matter [1]. Morphological and proliferative changes to neuroglia, particularly astrocytes, occur during the induction of disease and with neurodegenerative progression, originally defined as astrocyte reactivity (referring to pathological astrocyte hypertrophy) [1,2,3]. Despite the observable neuropathological reactive changes, morphological and functional modifications to these cells do not always correspond to each other, nor do the effects of the heterogeneous population of astrocytes that are exerted on other integrated cells throughout lesional and non-lesion areas. This heterotypic disease profile may relate to the existence of multiple astrocytic subgroups with different phenotypes that vary in responsiveness according to the stage of the disease and the regulatory processes that are governed by canonical and non-canonical signaling pathways [3].

Astrocytes of gray matter (protoplasmic astrocytes) contain a great number of shorter and highly branched outgrowths with end-feet that contribute to the formation of the blood–brain barrier (BBB) and the glia limitans [2]. On the other hand, white matter astrocytes (fibrous astrocytes) are characterized by fewer, longer, and less branched outgrowths [2,4]. The structural support provided by the astrocytic processes is based on cytoskeletal proteins such as the glial fibrillary acidic protein (GFAP) which constitutes a distinctive biomarker of astrocyte reactivity [5]. Astrocytic physiological function is integral for synaptogenesis, metabolic homeostasis, extracellular fluid maintenance, BBB integrity, and immunological regulation, to name but a few [4,5]. Transcriptional profiling has uncovered disparate messenger RNA and proteomic signatures, that include neurotransmitter receptors, in distinct astrocytic subpopulations at specific neuroanatomical regions (hippocampus, hypothalamus, amygdala, neocortex), supporting the adaptability and functional diversification of these subpopulations [4,5,6]. Consequently, astrocytes comprise a unique cell type capable of supporting neuronal survival dependent on regional physiological demands while also exerting further modulation on other neuroglial phenotypes to ensure integration with the regional neurons, such as dynamic myelination [1,2,4,5].

## 2. Astrocytes as Regulators of CNS Homeostasis and Metabolism

Astrocytes extensively influence oligodendrocyte lineage and neurons. They express high levels of glutamate transporters such as excitatory amino acid transporters 1 (EAAT1, also known as GLAST) and EAAT2 [7]. The accumulation of glutamate in the extracellular space is limited by the entry of glutamate into astrocytes and, thus, neurons and other glia cells are protected from excessive activation and excitotoxic cell death [8]. Furthermore, another basic function of astrocytes is the management of water, electrolytes, and pH, creating a suitable microenvironment for the survival, proliferation, and maturation of oligodendrocyte precursor cells (OPCs) and neurons [2]. In this context the importance of the glymphatic system emerges. The glymphatic system is created by astrocytic perivascular end-feet and is responsible for CNS interstitial fluid regulation and clarification [9]. Aquaporin 4 (AQP4) appears to be highly expressed in these astrocytic perivascular end-feet [10]. Genetic experiments in mice showed that the deletion of AQP4 increased interstitial fluid volume [11], while it has also been associated with cytotoxic edema formation during hypoxia or stroke [12]. Neuromyelitis optica spectrum disorder (NMOSD) constitutes a distinctive spectrum of autoimmune CNS diseases associated with the production of Immunoglobulin G (IgG) autoantibodies targeting AQP4 [13] and characterized by acute optic neuritis, transverse myelitis, and more scattered lesions (cerebral, cerebellar, brainstem) [14]. Astrocytes, with their AQP4-rich end-feet, are primarily affected by the autoantibody-mediated inflammatory response, including complement activation, white blood cells, CNS infiltration, and secondary oligodendrocyte damage leading to demyelination and axonal loss [15,16]. The existence of gap junctions between astrocytes and oligodendrocytes leads to the formation of a functional syncytium that supports the movement of intracellular metabolic substances from astrocytes to oligodendrocytes [1]. A further important function of this coupling is the dilution of increased K^+^ concentration in oligodendrocytes through direct passage into the astrocytic cytoplasm [17]. Finally, astrocytes release gliotransmitters such as glutamate, purine, and gamma-aminobutyric acid (GABA) (in response to neurotransmitters released from nearby synapses) into the synaptic cleft and thus can regulate neuronal excitability [18]. These regulatory molecules then respond to presynaptic neurons to modulate synaptic function [19]. The aforementioned role of astrocytes in synaptic activity has given rise to the concept of ‘tripartite synapse’, where astrocytes form an active, indispensable regulatory component of the synapse [20]. At the same time, the interactions of neurons and astrocytes with the BBB and extracellular matrix leads to the concept of the penta-partite synapse [21]. Since synaptic signal transmission can trigger astrocytes to secrete the leukemia inhibitory factor (LIF) [22], the support and maintenance of healthy signal transmission appears important for the regulation of myelination [1].

OPCs (or NG2 cells) are the main precursor cells, predominantly differentiating to mature oligodendrocytes, but also other glia types and neurons [1]. An interesting characteristic of OPCs is their reception of glutamate and GABA synapses from neurons, which perhaps control proliferation and differentiation [2]. Oligodendrocytes are responsible for myelination during CNS development and after a CNS insult leading to demyelination. The process of myelination advances with characteristic sequences, which are spatially and temporally determined [1,4]. In humans, after 16 weeks of gestation, myelin can be detected in the fasciculus cuneatus and, shortly after, appears in the pyramidal and cerebellar tracts. Then, during the first year, myelination proceeds swiftly in a cranial direction (from the occipital to the fronto-temporal lobes) [1,2]. Vital homeostatic regions appear to be myelinated before areas involved in more complex tasks such as the frontal cortex. Furthermore, late myelinated areas typically develop less myelination than early myelinating ones [3]. A lack of microenvironmental support seems to obstruct the adequate production of OPCs [1]. Lastly, studies have shown that Schwann cells can also briefly *stand in* for oligodendrocytes after a demyelination event before reactive astrocytes remove them [2]. Their possible roles consist of axon preservation and the production of trophic factors that support the survival of OPCs [1,5]. Astrocytes play important roles in OPC differentiation, survival, and proliferation by providing molecules such as platelet-derived growth factor (PDGF), fibroblast growth factor 2 (FGF-2), ciliary neurotrophic factor (CTNF), LIF, brain-derived neurotrophic factor (BDNF), and insulin-like growth factor 1 (IGF-1) (Table 1).

Cholesterol constitutes a fundamental molecule to every cell type in the body, as it is an important component of cellular membranes. Physiological brain development requires cholesterol as a precursor to many signaling molecules such as steroid hormones and, most importantly, as a major structural component of myelin sheaths [23]. The BBB impedes the transport of either nutritionally absorbed or hepatically synthesized cholesterol and, as a result, cholesterol must be de novo synthesized within the CNS [24]. Astrocytes are proposed to be one of the primary cellular sources of cholesterol [1,25], which is conveyed to oligodendrocytes by means of lipoproteins containing apolipoprotein E [26]. Homeostatic alterations in astrocytes can also be seen in MS models such as experimental autoimmune encephalomyelitis (EAE) [27,28] and in the cuprizone-induced model through the recruitment of microglia [29]. Gene expression studies in astrocytes during the later stages of EAE unveil that the most frequent expression alterations concern the genes involved in cholesterol synthesis, such as the sterol regulatory element-binding (SREB) cleavage-activating protein (SCAP), an essential co-activator of the transcription factor SREB [30]. Decreases in cholesterol metabolism genes are associated with increases in genes involved with immune pathways [31] creating an inhibitory milieu for remyelination. 

An essential supportive role is played by astrocytes during hypoglycemia, as well as during the period of increased neural activities in the supply of energy, which is important for myelination. This energy is produced from glucose after its entry via the BBB and its intracellular transportation by glucose transporters (GLUT-1) in the perivascular end-feet [32] or from gluconeogenesis/glycogenolysis processes, since these are limited to astrocytes [33]. Astrocytes under glucose inadequacy can break stored glycogen into lactate and thus increase the participation of lactate to energy metabolism of nearby axons when such deprivations occur [34]. Oligodendrocytes express high levels of monocarboxylate transporter 1 (MCT-1) and, as a result, consume lactate at higher levels than other CNS cells to produce myelin by lipogenesis. Consequently, some astrocytic lactate may head towards oligodendrocytes to facilitate myelination [35]. Moreover, in accordance with the hypothesis of astrocyte—neuron lactate transfer shuttle (ANLTS), it has been found that not only can lactate be distributed directly from astrocytes to neurons at the nodes of Ranvier [33], but also that oligodendrocytes are a potential source of lactate to support axon function [36].

## 3. Astrocyte Subgroups: Moving Away from the A1-A2 Dipole

The level of GFAP expression reflects the continuum of morphological modifications of reactive astrocytes [4,5]. Initial studies defined a dual astrocytic response upon CNS injury with “mild” astrogliosis (referred to as activation) being related to CNS restoration and protection, while “severe” astrogliosis (or reactivity) was associated with the prevention of CNS repair and glial scar formation [30,37,38,39,40]. Despite astrocyte hypertrophy being characterized by increased GFAP expression, it has little specificity, and the increase does not always correspond to disease severity, so a combination of astrocytic biomarkers (aldehyde dehydrogenase-1, glutamine synthetase, and aldolase-C) is required for a better characterization of the pathological astrocytosis [3]. Furthermore, recent RNA sequencing analyses demonstrated significant heterogeneity among astrocytes, describing different molecular profiles of normal and reactive subgroups in separate anatomical areas and during CNS disease outcomes [3,5]. 

More specifically, reactive astrocytes from different subcortical MS lesions showed increased GFAP, CD44 molecules, and FOS and BLC6 transcription factors [41]. Another subset is characterized by the increase in granulocyte-macrophage colony-stimulating factor (GM-CSF) and the nuclear factor erythroid 2-related factor 2 (NRF2) decrease caused by increased MAF BZIP transcription factor G (MAFG) [42]. Additionally, another study revealed further heterogeneity among relapsing–remitting and progressive forms of MS with decreased antioxidant genes expression and increased expression of complement factor 3 (C3) in the astrocytes of the former [43]. Finally, an astrocyte subgroup with anti-inflammatory functions was detected by the expression of the lysosome-associated membrane glycoprotein 1 (LAMP1) and tumor-necrosis-factor-related apoptosis-inducing ligand (TRAIL) [44] (Table 2).

Early studies pointed towards a dipole of reactive astrocytes, with distinct genetic signatures, called A1 and A2 [6,30,37,45]. The A1 type was considered noxious, induced by activated microglia and producing proinflammatory molecules such as interleukin 1 (IL1) and tumor necrosis factor α (TNFα), which can abrogate the differentiation and proliferation of OPCs [6,40,46]. On the other hand, the A2 type, induced by ischemia, was considered neuroprotective [6,45]. Nevertheless, studies in mice and from human archival tissues exhibiting primary and secondary neurodegeneration (Alzheimer’s disease, Huntington’s disease, and MS) may suggest an alternate nomenclature to the aforementioned phenotyping [3]. Specifically, reactive astrocytes express only a portion of the A1/A2 gene markers, while the function of many gene products remains unexplored and, thus, no clear protective or deleterious effects can be attributed to them [3,6]. Finally, assorted molecular profiles have emerged, many of which depend on the disease stage [3].

## 4. Astrocytes and Aging

Aging constitutes the natural process of gradual tissue dysfunction and degeneration affecting various organs and systems, including the CNS. Aging is also characterized as the main risk factor for the development of neurodegenerative diseases. Possible mechanisms of aging encompass cellular senescence which—as a main mechanism—interacts with stem cell exhaustion, telomere attrition and genomic instability, oxidative stress, epigenetic modifications, and intercellular-signaling dysfunction-promoting aging degeneration [47]. The CNS is considered to be one of the most age-resilient systems of the human body, possibly reflecting its phenomenal plasticity [48]. Despite that, brain parenchymal atrophy is a main feature of aging [49]. The effect of aging on neuroglia varies with oligodendrocytes and microglia—particularly those of the white matter [50]—being the most affected, while the effect on astrocytes is relatively milder [5,48,49]. In terms of astrocytic aging there are two main processes [40]. On the one hand, astrocytes go through replicative senescence, which constitutes a decrease in cellular proliferation with increasing cell divisions [51]. On the other hand, astrocytes experience stress-related senescence when exposed to reactive oxygen species (ROS) and other stress-inducing factors [52]. In both cases, an increase in senescence markers, such as senescence-associated β-galactosidase, p16, p21, and p53, was displayed [53]. However, studies in mice demonstrated diverse and often conflicting alterations with atrophy, astrocyte depletion, and hypertrophy, with increased levels of GFAP (closely resembling the A1 reactive phenotype) [48,50,54]. This age-associated reactivity is thought to be induced by cytokines produced by activated microglia which, in turn, become activated because of an increased clearance of age-related myelin fragments [49,55,56]. On the molecular level, astrocytic age-dependent alterations include a decrease in glutamate and purinergic receptors [57], AQP4 channels, and the derangement of astrocytic vesicles [58]. At the same time, there is upregulation of numerous immunity-related genes [50]. Firstly, the increased expression of major histocompatibility complex (MHC)-I [59] augments the antigen-presenting capability of astrocytes leading to a deleterious pro-inflammatory state [60] or to the protective scour of age-related myelin fragments [61]. In addition, there is an increased production of proinflammatory factors such as chemokine CXC motif ligand 10 (which promotes T cell chemoattraction), CXCL5 (a neutrophil chemoattractant), and complement factors C3 and C4b, while the production of neurotrophic factors such as BDNF and energy metabolism diminish [62]. Finally, the disruption of cholesterol synthesis—a key molecule for myelin formation by oligodendrocytes and thus neuronal function—because of age-induced 3-hydroxy-3-methylglutaryl coenzyme A (HMGCoA) reductase downregulation could affect CNS function [63]. The previously described morphological and molecular changes differed between brain regions and mostly affected vulnerable areas associated with neurodegenerative pathologies (such as the hippocampus), indicating local differentiation in gene expression [50,54,64]. This increased adoption of the A1-reactive phenotype could therefore participate in the development of neurodegenerative diseases [65]. Despite this, the effects of aging have yet to be sufficiently described to confidently confirm a reactive astrocytic phenotype.

## 5. The Astrocytic Response during Multiple Sclerosis

**Figure 1:**
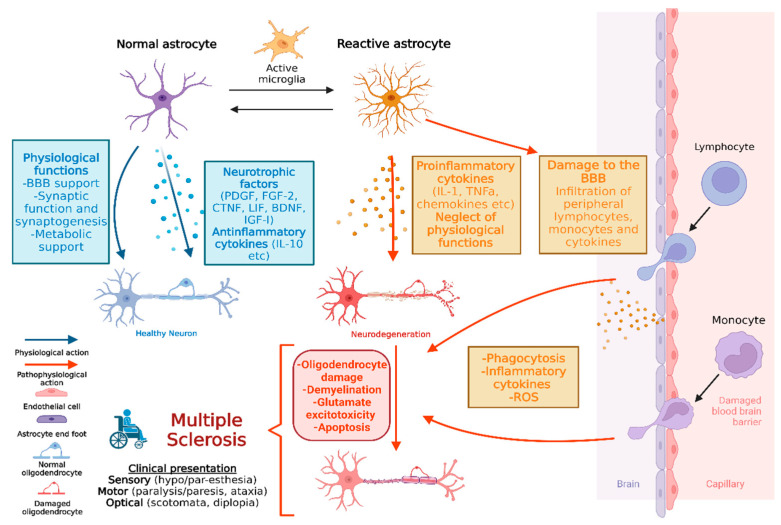
The multifaceted role of astrocytes in healthy conditions and multiple sclerosis (created with Biorender).

MS constitutes an inflammatory autoimmune demyelinating disease of the CNS, where there are two main forms: (1) relapsing-remitting, with the inflammatory element predominating, gradually leaving neurological deficits; and (2) the progressive form, with either a primary continuous and mainly degenerative course with a poor prognosis, or a secondary neurodegenerative transition following a protracted period of relapse [5]. However, it has recently been argued that MS can be more accurately defined as a continuum with variable pathophysiological outcomes operative across the duration of the disease in individuals that are impacted in accordance to their specific genetic and environmental risk factors, along with the capacity to compensate for these through plasticity and remyelination [66]. Regarding the classical clinical presentation, it can vary with sensory (paresthesia, hypoesthesia), motor (paresis, spasticity, ataxia, and tremor), orthostatic, and visual (scotomata, diplopia) disorders. As for the pathological characteristics of MS, they include inflammation, demyelination, axonal/neuronal damage, and reactive astrogliosis. Reactive astrocytes take part in the pathogenesis of MS, influencing both oligodendrocytes and neurons [2,5]. The effects of reactive astrocytes on neurons include both the production of pro-inflammatory/neurotoxic factors and the loss of normal supportive functions. More specifically, astrocyte activation by raised BDNF levels leads to increased nitric oxide production which acts as a reciprocating transmitter causing neurotoxicity [67]. Additionally, astrocytic IL-6 and granulocyte-macrophage colony-stimulating factor (GM-CSF) production stimulates pro-inflammatory microglia, displaying the importance of bidirectional crosstalk between different CNS cell types [68]. Furthermore, the loss of normal functions, such as glutamate uptake and potassium buffering, as a result of the diminished expression of their respective transporters, such as the excitatory amino acid transporters (EAATs) and inward rectifying K+ channels, disorganizes the neuronal environment and induces excitotoxicity [69]. Lastly, the derangement of the aforementioned astrocytic metabolic provision of lactate and cholesterol also assists neurodegeneration [70]. Furthermore, reactive astrocytes produce a plethora of factors which influence oligodendrocyte and OPCs, as mentioned above (Table 1). The distinctive MS plaques are classified into active (acute), chronically active (inactive center with active periphery, both characteristics of relapsing–remitting form), inactive (chronic, characteristic of primary progressive form), and early (local perivascular inflammation with BBB breakdown) [4]. Regarding active plaques, there are hypertrophic astrocytes with extended branches rich in GFAP [4,64]. At the molecular level, there is an increased expression of GFAP and other structural proteins (such as vimentin) as well as an increased production of inflammatory molecules and growth factors such as BDNF, CTNF, and IGF-1 [71,72]. In addition, elevated levels of glutamate are found (indicative of changes in its transport mechanisms) causing excitotoxicity [73]. The inactive plaques appear to contain milder reactivity with smaller and fewer branched astrocytes surrounding degenerated axons [4,5]. Finally, in chronically active plaques, peripheral inflammation and demyelination envelop the central oligodendrocytes to prevent remyelination [74] (see Figure 1).

## 6. Established Therapies for Multiple Sclerosis

The current treatment for MS is based on three main objectives: the treatment of relapses, symptomatic control, and disease-modifying therapies (DMTs) which primarily target the adaptive immune system. Most of the available therapeutic options are focused on the relapsing–remitting form, while only ocrelizumab (a monoclonal antibody targeting CD20+ B cells) has been approved for the primary progressive disease [75,76]. 

The treatment of relapses is based on the administration of steroids due to their anti-inflammatory effect (stabilization of the BBB, edema reduction, and immune cell suppression). Methylprednisolone is the main steroid used, either intravenously or *per os* [77]. More drastic measures in special cases include plasmapheresis to remove inflammatory factors or intravenous immunoglobulin, which targets the autoreactive B cells and the autoantibodies that may be present against myelin [78].

Regarding DMTs, a variety of immunomodulatory agents are included (Table 3). First-line drugs encompass interferons, glatiramer acetate, teriflunomide, and dimethyl fumarate. Interferons (IFNβ-1a, IFNβ-1b, and pegylated IFNs with a better pharmacokinetic profile) suppress the production of proinflammatory cytokines (such as IL-17) and increase the anti-inflammatory and neurotrophic factors (such as IL-10 and nerve growth factor; NGF) [79]. Glatiramer acetate, which structurally resembles myelin basic protein (MBP), displaces MBP from the MHC-II molecules, altering the immune response towards type 2 T-helper (Th2) response with the production of anti-inflammatory cytokines [80]. Teriflunomide, acting as a pyrimidine synthesis antagonist, decreases the proliferation of active T and B cells, suspending their participation in MS pathogenesis [81]. Finally, dimethyl fumarate, which operates by an unknown mechanism related to the signaling pathway of the transcription factor, nuclear factor erythroid 2-related factor 2 (Nrf2), increases the antioxidative defenses, such as elevated glutathione turnover in astrocytes, oligodendrocytes, and neurons, preventing cell death [82].

On the other hand, second-line drugs include sphingosine 1-phosphate receptor (S1PR) modulators, Cladribine, and Mitoxantrone. S1PR modulators such as Fingolimod, Siponimod, Ozanimod, and Ponesimod bind to their respective receptors on T, B, and natural killer (NK) cells, inhibiting lymph node exit and thus reducing entry into the CNS and participating in MS inflammation [83]. Cladribine, a synthetic purine analog, depletes T, B, and dendritic cells by disturbing their DNA metabolism, causing DNA damage and apoptosis [84]. Mitoxantrone, which is an immunomodulatory and antineoplastic agent, interferes with DNA synthesis, causing the suppression of T and B cells and inhibiting the secretion of inflammatory cytokines IL-2 and IFN-γ [85].

Finally, monoclonal antibodies are steadily gaining ground in the treatment of MS, targeting surface antigens of lymphocytes and other white blood cells (macrophages, dendritic cells, and NK cells). Natalizumab, the first human monoclonal antibody used in relapsing–remitting MS [86], binds to integrin a4, obstructing the passage of cells through the BBB [79,86]. Alemtuzumab is a humanized monoclonal antibody against the CD52 molecule on the surface of T, B, and other cells [87], and the corresponding antibody ocrelizumab targets the CD20 size on the surface of B cells, leading to their destruction by cell- and complement-dependent mechanisms [88].

## 7. Astrocyte-Selective Therapies: Challenges and Potential Solutions

The development of treatments targeting astrocytes constitutes a key goal of studying astrocytic subsets. In this context, the challenges are to elucidate the role of astrocytes in disease pathogenesis and design disease-specific therapies [75]. The characterization of the heterogeneous astrocyte subgroups, through the identification of sets of characteristic molecular markers for each subgroup, may provide a targeted approach in the pursuit of limiting the neurodegenerative changes associated with CNS inflammation. Using a combination of RNA sequencing, immunohistochemistry, and functional assay techniques specific to astrocytes, in combination with specific or conditional gene knockout models and CRISPR/Cas gene editing, will advance our understanding about the astrocytic phenotypes that mainly contribute to neuroinflammatory lesion evolution and may provide clues for their modulation [3]. For example, transcriptional and metabolomic studies in models of MS such as experimental autoimmune encephalomyelitis (EAE) have demonstrated that the production of a sphingolipid (galactosylceramide) activates phospholipase A2 [89]. The interaction between a structural domain of phospholipase A2 and the mitochondrial antiviral signaling protein (MAVS), on one hand, enhances the pro-inflammatory actions of astrocytes while, on the other hand, deactivates hexokinase 2 and thus interrupts the production of lactate [70], which is a key energy metabolite of neurons and oligodendrocytes [1,30]. Miglustat, a pharmacologic agent approved for sphingolipidoses (Gaucher I disease, Niemann–Pick disease) inhibits the synthesis of glucosylceramide and, as a result, the synthesis of galactosylceramide appeared to reduce the astrocytic inflammatory response inhibiting the progression of EAE [70,90]. Furthermore, in models of EAE, and in archival tissue from individuals that lived with MS, it could be demonstrated that the expression of antioxidant protein peroxiredoxin 6 was induced in reactive astrocytes [91], with consequently limited the BBB breakdown, immune cell entry, and activation of microglial cells, mitigating inflammation and demyelination [92]. In this context, more specific drugs could be developed that induce or suppress the above molecular drivers of pathogenesis or corresponding metabolic-inflammatory signaling pathways of specific “damaging” or “protective” astrocyte subsets that participate in MS progression. In addition, it is important to carefully select appropriate models for each disease by studying human samples to avoid morphological and functional differences with experimental animal models of MS-like disease [75,79], while the complex interactions between astrocytes and the rest of the CNS cells should not be overlooked. Finally, a new and promising area of research in the management of MS is extracellular vesicles/exosomes (EVs). A major clinical utility of EVs may be as disease-specific biomarkers, as their levels and content (proteins and nucleic acids) are related to the activity and may serve as a pathognomonic feature of MS, enabling the monitoring of the disease [93]. On the other hand, EVs may be utilized in the treatment of MS initially, as indicators of patient-specific responses to existing immunomodulating treatments, as studies have shown that they are affected by them [94]. Ultimately, they may be used as therapeutic agents for delivering neuroprotective growth factors to the autoimmunity-modifying antigens (myelin oligodendrocyte glycoprotein; MOG, proteolipid protein; PLP, and MBP) which promote immune tolerance and constitute the cutting-edge technology of “extracellular vesicle-based vaccines” [93,95].

## 8. Conclusions—Future Remarks

Many studies have demonstrated the importance of astrocytes in the physiological function of the CNS. This review highlights the diversity of astrocyte responses to injury due to different reactive subsets. In particular, the role of reactive astrocytes in MS is multidimensional and complex. Diverse astrocytic subgroups in distinct regions of the CNS, and at different phases of the disease, positively (anti-inflammatory phenotypes) or negatively (pro-inflammatory phenotypes) influence disease progression. Beyond the wide range of established and recent immunomodulatory therapies that mainly target the relapsing–remitting form of the disease, there is an unmet medical need for the treatment of the progressive forms. Astrocytes could be a new therapeutic cell-specific target, as they actively participate in the development of the disease and interact with a multitude of other pathogenically active cells. Therefore, more studies are needed for the molecular identification and understanding of the role of various astrocytic subpopulations throughout the course of MS in an attempt to develop targeted therapies.

## Figures and Tables

**Table 1:** Astrocyte-derived molecules and their effect on oligodendrocyte precursor cells (OPCs) and oligodendrocytes.

Molecule	Effects
BDNF	Promotion of remyelination
CTNF	Enhancement of OPC migration, protection from apoptosis and decrease myelin destruction
FGF2	OPCs proliferation, disruption of OPC differentiation (in combination with PDGF)
PDGF	Proliferation and survival of oligodendrocytes and OPCs migration
LIF	Survival, differentiation and maturation of oligodendrocytes
IL-1	Induction of apoptosis and obstruction of myelination
TNFα	Inhibition the differentiation and proliferation of OPCs
IFNγ	Reduction in OPCs proliferation, promotion of OPCs survival, and differentiation through induction of PDGF
IL-6	Reduction in OPCs differentiation
CXCL1	Inhibition of OPCs migration

**Table 2:** Astrocyte subpopulations.

Subpopulation Type	Markers
Pro-inflammatory	CD44, FOS, BLC6
Pro-inflammatory	NRF2, MAFG, GM-CSF
Pro-inflammatory	C3
Anti-inflammatory	LAMP1, TRAIL

**Table 3:** Established multiple sclerosis disease-modifying therapies.

Drug Class	Active Substance	Mechanism of Action
Interferons	IFNβ-1a, IFNβ-1b, pegylated IFNs	Increase in anti-inflammatory and trophic factors
Immunomodulators	Glatiramer acetate	Alteration of immune response towards the anti-inflammatory T helper 2 type
	Teriflunomide	Inhibition of active T and B lymphocytes proliferation
	Dimethyl fumarate	Unknown mechanism, possible enhancement of oxidative stress protection
Sphingosine 1-Phosphate (S1P) Receptor (S1PR) modulators	Fingolimod, Ozanimod, Siponimod	Prevention of T, B and NK cells lymph node mobilization
Monoclonal antibodies	Natalizumab	Blockage of CNS infiltration
	Alemtuzumab	Depletion of T and B cells by targeting CD52 surface molecule
	Ocrelizumab	Depletion of B cells by targeting CD20 surface molecule
Cell-depleting agents	Cladribine	Depletion of T, B cells and dendritic cells by targeting DNA metabolism
	Mitoxantrone	Depletion of T, B cells and dendritic cells by targeting DNA metabolism and anti-inflammatory action

## Data Availability

Not applicable.

## Abbreviations

ALS	Amyotrophic lateral sclerosis
IGF-1	Insulin-like growth factor 1
GFAP	Glial fibrillary acidic protein
TNFα	Tumor necrosis factor a
BDNF	Brain-derived neurotrophic factor
CTNF	Ciliary neurotrophic factor
DMTs	Disease-modifying therapies
MBP	Myelin basic protein
NGF	Nerve growth factor
EAE	Experimental autoimmune encephalitis
CRISPR	Clustered Regularly Interspaced Short Palindromic Repeats
MAVS	Mitochondrial antiviral signaling protein
MOG	Myelin oligodendrocyte glycoprotein
PLP	Proteolipid protein
MCT-1	Monocarboxylate transporter 1
GLUT-1	Glucose transporter 1
SREBP	Sterol regulatory element-binding protein
EAAT	Excitatory amino acid transporter
GLAST	Glutamate/aspartate transporter
